# Crowdsourcing Awareness: Exploration of the Ovarian Cancer Knowledge Gap through Amazon Mechanical Turk

**DOI:** 10.1371/journal.pone.0085508

**Published:** 2014-01-22

**Authors:** Rebecca R. Carter, Analisa DiFeo, Kath Bogie, Guo-Qiang Zhang, Jiayang Sun

**Affiliations:** 1 Department of Epidemiology and Biostatistics, School of Medicine, Case Western Reserve University, Cleveland, Ohio, United States of America; 2 Case Comprehensive Cancer Center, Case Western Reserve University, Cleveland, Ohio, United States of America; 3 Departments of Orthopaedics and Biomedical Engineering, Case Western Reserve University, Cleveland, Ohio, United States of America; Cleveland Clinic Lerner Research Institute, United States of America

## Abstract

**Background:**

Ovarian cancer is the most lethal gynecologic disease in the United States, with more women dying from this cancer than all gynecological cancers combined. Ovarian cancer has been termed the “silent killer” because some patients do not show clear symptoms at an early stage. Currently, there is a lack of approved and effective early diagnostic tools for ovarian cancer. There is also an apparent severe knowledge gap of ovarian cancer in general and of its indicative symptoms among both public and many health professionals. These factors have significantly contributed to the late stage diagnosis of most ovarian cancer patients (63% are diagnosed at Stage III or above), where the 5-year survival rate is less than 30%. The paucity of knowledge concerning ovarian cancer in the United States is unknown.

**Methods:**

The present investigation examined current public awareness and knowledge about ovarian cancer. The study implemented design strategies to develop an unbiased survey with quality control measures, including the modern application of multiple statistical analyses. The survey assessed a reasonable proxy of the US population by crowdsourcing participants through the online task marketplace Amazon Mechanical Turk, at a highly condensed rate of cost and time compared to traditional recruitment methods.

**Conclusion:**

Knowledge of ovarian cancer was compared to that of breast cancer using repeated measures, bias control and other quality control measures in the survey design. Analyses included multinomial logistic regression and categorical data analysis procedures such as correspondence analysis, among other statistics. We confirmed the relatively poor public knowledge of ovarian cancer among the US population. The simple, yet novel design should set an example for designing surveys to obtain quality data via Amazon Mechanical Turk with the associated analyses.

## Introduction

Ovarian cancer is the most deadly gynecologic malignancy in the United States and the fifth leading cause of cancer death in women. According to the American Cancer Society's 2013 estimate, ovarian cancer is predicted to cause 22,240 new diagnoses with 14,030 deaths in the United States alone [1]. The overall 5-year survival rate for all ovarian cancer patients is 43.7% compared to 89% for all breast cancer patients. Specifically, 63% of ovarian cancer malignancies are late stage diagnoses, causing patients at this stage to experience a 5-year survival rate of only 26.9%, according to the American Congress of Obstetricians and Gynecologists (ACOG) [2]. This poor survival rate is largely due to the lack of effective, approved ovarian cancer screening tools, in contrast to the existence of mammograms and PSA tests for the screening of breast cancer and prostate cancer, respectively. Historically, ovarian cancer is known as “the silent killer,” because some patients do not show disease-specific symptoms for ovarian cancer at an early stage [3]. Known symptoms include bodily changes reflected by feeling full quickly, pelvic pain, sudden weight gain or weight loss, frequent urination, bloating around the midsection, fatigue, back pain, prolonged menstrual cycles or bleeding, and fluid in the stomach [3]. In fact, more than 80% of ovarian cancer patients actually showed symptoms, even while the disease was still limited to the ovaries [4]. However, some of these symptoms can be misdiagnosed or dismissed by both patients and even some health professionals. There is a critical knowledge gap amongst both the general public and practicing physicians concerning ovarian cancer and its indicative symptoms [5].

### Promotion of Survivorship with Knowledge

Cancer survival has improved during the last few decades, especially for patients diagnosed with breast cancer, the most common cancer among women. New and better combinations of treatments and screening tools for breast cancer have extended and improved the lives of survivors [6], [7]. Visibility and spread of knowledge has been spearheaded by international awareness campaigns [8]–[10]. Social campaigns in particular, such as National Breast Cancer Awareness Month (NBCAM) have been highly successful in increasing routine screening within two years to approximately 70% of the general female population and contributed to substantial reductions in breast cancer mortality [8], [11]–[13]. Unfortunately, ovarian cancer does not currently benefit from a widespread media-fueled awareness campaign. The lack of awareness potentially generates the majority of late stage diagnoses and consequent low survival rate. The purpose of the current study is to assess and confirm this critical knowledge gap, thereby highlighting the need for increased awareness towards the disease. Increased public awareness and a broader spread of knowledge are expected to promote early diagnoses rates and additional research in ovarian cancer, following the model successfully accomplished for breast cancer.

### Assessment of the U.S. Population through Web-Based Surveys

To identify a knowledge gap, it is advisable to start by collecting data via a survey of the US population. Surveys typically can be done in two ways: a traditional paper-based survey or a web survey. Ensuring a representative sample is often a challenge in traditional survey study design. The overall goal is to achieve generalizable results, however this can entail economic obstacles due to budget, time, and manpower requirements to meet study needs [14]. Result validity, which is impacted by response rates, data-entry, and analyses, must also be considered. In the past decade, web-based surveys have been developed as a credible means of collecting data from large sample groups quickly and at minimal cost [15]. In 2004 a consortium of researchers from University of Texas at Austin, Stanford, and University of California, Berkeley addressed several concerns about data collection using the Internet, such as sample diversity, generalization, and reproducibility. In a comparison of a very large Internet sample (N = 361,703) with 510 published traditional samples, the consortium determined the Internet sample to be more diverse in demographics such as gender, socioeconomic status, geographic region, and age than traditional samples. They concluded that web-questionnaire results generalize across various survey formats, do not appear to be tainted by false data or repeat responders, and are consistent with results that use good traditional methodologies [16]. A 2012 study by Greenlaw et al. confirmed these earlier conclusions by comparing web-based and paper-based survey methods [14]. Specifically, the authors stated that there was “overwhelming support” for the cost-effectiveness and validity of web-based survey administration in comparison to traditional methods, noting the “marked reduction” of the overall cost per response as well as the effort necessary to produce and distribute surveys online.

### Use of Online Crowdsourcing to Assess a Proxy of the U.S. Population

Successful surveys need to possess two important characteristics: 1) an unbiased design and 2) an excellent recruitment strategy. In our study, for 1) we designed an effective survey specifically tailored for online surveys to have both a quality control element and unbiased data, as well as good participation. For 2) we took advantage of modern crowdsourcing through the relatively new platform Amazon Mechanical Turk. The platform, released by Amazon.com in 2005, facilitates the design, dissemination, storage of data, and data analysis of web-based surveys. Amazon Mechanical Turk, hereafter referred to as **MTurk**, is a digital marketplace for work through which users can perform online “tasks” in exchange for a nominal fee. Employees (called *workers*) are recruited by employers (called *requesters*) for the execution of tasks, (known as Human Intelligence Tasks, or *HITs*). Both workers and requesters are anonymous and harness MTurk's utilities through a unique ID provided by Amazon. A requester can accept or reject results submitted by a worker, controlling whether a worker is paid or not. Data is compiled from the website into a downloadable Excel spreadsheet for analysis. The advantages of MTurk are well documented [17]. Workers tend to be from a diverse background, spanning a wide range of age, ethnicities and socioeconomic status [18]. Researchers have verified that MTurk demographic responses are accurate [19]. Furthermore, their psychometric properties are valid [20] and replicable [21]–[23].

In the current study, we designed a simple yet innovative survey completed at an accelerated rate of time and reduced cost compared to traditional recruitment methods. Breast cancer was chosen as the control group to compare with awareness of ovarian cancer in a representative crowdsourced sample of online respondents (See: 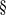
2 Methods). Our crowdsourcing of workers, recruited through Amazon Mechanical Turk, reflected a reasonable proxy of the US population at a minimized rate of cost and time (See: Data Collection in 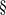
3). We investigated the features of awareness of ovarian cancer among the sample (See: 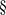
3 Analyses and Results). We showed that workers consistently present a lack of awareness of ovarian cancer impact or significance. Finally, we demonstrated that in addition to presenting a lack of of ovarian cancer awareness, the worker's explicit knowledge of ovarian cancer varied widely, above and beyond simply guessing (See: 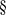
4 Discussion & Conclusions).

## Methods

### Design

We devised our survey with 1) an experimental control element, 2) a two-stage quality control mechanism, 3) repeated measures, and 4) additional quality control steps. The study design included basic awareness questions about ovarian cancer (using breast cancer as the control group), as listed in Table 1 (Questions 1–11), repeated measures (Questions 12–15), and additional quality control mechanisms (Questions 7a–11a) to avoid confounding factors and delineate between quality responses and possible guesses. Other quality control measurement elements consisted of pricing, timing, clear and concise user instructions, and inclusions/exclusion criterion (See: Procedure).

**Table 1:** Survey Questions and Descriptive Statistics.

Variable (Abbreviation)/Question	Response	No. (%)
**1. Age (age)**	18–30 Years	121 (60)
How old are you?	31–40 Years	37 (18)
(Complete distribution is shown in Figure 1)	41–50 Years	24 (12)
	51–61 Years	20 (10)
**2. Gender (gender)**	Male	115 (57)
	Female	87 (43)
**3. Knowledge about breast cancer (kbrc)**	**Very Well**	**22 (11)**
How well do you know about breast cancer?	Fairly Well	137 (68)
	Not at All	43 (21)
**4. Knowledge about ovarian cancer (kovc)**	**Very Well**	**5 (2)**
How well do you know about ovarian cancer?	Fairly Well	84 (42)
	Not at All	113 (56)
**5. Background about breast cancer (bbrc)**	**Yes**	**92 (46)**
Do you know anyone CLOSE TO YOU who has been diagnosed with breast cancer?	No	110 (54)
**6. Background about ovarian cancer (bovc)**	**Yes**	**24 (12)**
Do you know anyone CLOSE TO YOU who has been diagnosed with ovarian cancer?	No	178 (88)
7^*^. Risk of breast cancer (riskbrc)	1∶8	76 (38)
What is a woman's lifetime risk of developing breast cancer?	1∶70	62 (31)
*The correct answer is 1∶8*	1∶200	57 (28)
**#** This Q is repeated (**riskbrcr**)	1∶1000	7 (3)
8* Risk of ovarian cancer (riskovc)	1∶8	21 (11)
What is a woman's lifetime risk of developing ovarian cancer?	1∶70	75 (37)
*The correct answer is 1∶70*	1∶200	49 (24)
**#** This Q is repeated (**riskovcr**)	1∶1000	57 (28)
9^*^. Color of breast cancer ribbon (colorbrc)	Teal	2 (1)
What color is the breast cancer awareness ribbon?	Pink	197 (97.5)
*The correct answer is Pink*	Red	2 (1)
**#** This Q is repeated (**colorbrcr**)	Orange	1 (0.5)
10^*^. Color of ovarian cancer ribbon (colorovc)	Teal	114 (57)
What color is the ovarian cancer awareness ribbon?	Pink	25 (12)
*The correct answer is Teal*	Red	39 (19)
**#** This Q is repeated (**colorovcr**)	Orange	24 (12)
11^*^. Knowledge on Lethality (lethal)	Breast	34 (16)
Which cancer is more lethal than the other? Breast, Ovarian, or Same?	Ovarian	84 (42)
*The correct answer is ovarian cancer*	Same	84 (42)

There are 11 primary questions. Questions 7 through 11 are augmented with a follow-up self-assessment of how sure an individual is of their answer (indicated here by * and in the actual survey as Questions 7a–11a). This will provide an independent assessment of their choice certainty, confirmed via statistical analysis. Questions 7 through 10 have been augmented as repeated measures (indicated here by **#** and duplicated in the actual survey as Questions 12–15). These duplicated questions' answer order was permuted in order to address knowledge of disease risk and ribbon color. Results were visualized correspondence analysis in Figure 4.

The justification of the design is as follows. First, simple awareness questions are quick and easy proxies to address the critical knowledge gap. Logically, if there is a severe gap in disease awareness, then there will be a severe gap in specific knowledge concerning symptoms and lifetime risk of diagnosis. Second, a control group and sound recruitment strategy specifically targeting a diverse population via the internet are the key elements in constructing a modern unbiased survey. Third, repeated questions with multiple choices arranged in a permuted order is an excellent way to assess how sure a participant is of their given answer, independent of his or her self-report of certainty in the answer. Fourth, some internet users may have a tendency to check answers to particular survey questions, therefore biasing survey results. Accordingly, clear user instructions and other quality control measures are necessary to discourage users from checking their answers and to derive a sound study.

### Survey Questions

The survey began with basic demographic questions to assess age and gender. Baseline ovarian cancer knowledge was addressed by workers determining how well they knew of ovarian cancer on a 3-point Likert scale of “Very well,” “Fairly Well,” or “Not at All.” To determine personal background and impact of ovarian cancer, workers were asked if they knew anyone close to them who had been diagnosed with ovarian cancer using a dichotomous Yes/No scale. Workers demonstrated their specific knowledge of a woman's lifetime ovarian cancer diagnosis risk by selecting from the ratio options of “1∶8,” “1∶70,” “1∶200,” and “1∶1000.” Public visibility of ovarian cancer was assessed through national cancer campaign ribbon color knowledge, where workers were asked, “What color is the ovarian cancer awareness ribbon?,” with the categorical options of “Teal,” “Pink,” “Red,” and “Orange.” Last, participant estimates of comparative ovarian cancer lethality were elicited with the question of, “Which cancer is more lethal than the other?,” where workers were provided the categorical options of “Breast,” “Ovarian,” or “Same.”

An experimental control element was added to the questionnaire by replicating the ovarian cancer awareness questions for breast cancer awareness. Breast cancer was chosen as a control measure, given that the disease is the most commonly diagnosed invasive cancer in women and its advocacy efforts have greatly increased public attention to breast cancer [24]. To facilitate this control element, workers were assessed on their baseline knowledge of breast cancer first, and then subsequently assessed using the same question format on their baseline knowledge of ovarian cancer. The survey assessed workers on their knowledge of someone close to them with breast cancer, their estimations of a woman's lifetime breast cancer risk, knowledge of the breast cancer awareness ribbon color, and comparative lethality of breast cancer.

We also sought to quantify the relative certainty of workers' responses [25]. For both the control element of breast cancer and the test element of ovarian cancer, we asked workers an additional question directly: “How sure are you of the accuracy of your answer to the previous question?” (See Questions 7a to 11a). Workers were offered two response options: that they were “50% sure,” implying they guessed, or “100% sure,” implying absolute confidence in their previous answer. The purpose of assessing uncertainty was two-fold. One, we wanted quality control assurance; to check that workers did not cheat by using a web search engine to inform their answers. Specific details of lifetime risks of various cancers are not necessarily at the forefront of public knowledge, therefore an overwhelming number of correct responses concerning statistical knowledge of either breast or ovarian cancer would alert us to a biased survey sample. Second and more importantly, an uncertainty component in the questionnaire would provide insight into the stability of the worker's responses. Namely, it would provide a valuable opportunity to evaluate the variability of the workers questionnaire choices, and a secondary check if the correct answer was a pure guess. This was relevant to the awareness knowledge we wanted to assess.

Last, a repeated measures element was included with the cancer awareness survey [26]. The purpose of the repeated measures element was to both assess for response variance independent of the workers' self-report of certainty and to prevent bias. The repeated measure consisted of duplicating Questions 7 through 10, and permuting the order of the original response options. This avoided positioning biases, meaning that workers would not be influenced by the original question's position among the offered selections [27]. By randomizing the response order of the repeated measures, we could also be sure if workers chose the option closest to their true knowledge with a consistent answer implying a personally-held belief, and an inconsistent answer implying the opposite [25].

### Procedure

The procedures to conduct a survey within MTurk are well-described [28]. For the present study, we initiated a survey using MTurk, after previously establishing an account and placing funds into the account where a 10% surcharge was assessed on all payments. The survey was uploaded on the MTurk website using the provided HTML editor, which functioned as a rudimentary webpage with the capacity to incorporate images, tables, figures, or videos. We then posted a job listing, or HIT, on the MTurk forum entitled “Cancer Awareness Survey.” The short survey was advertised as taking up to 10 minutes, with a payout of $0.40 cents per fully completed survey.

The payout, or monetary incentive, was a key design component where the pay rate needed to be fair by MTurk standards to encourage a higher rate of recruitment without discouraging the more discriminating workers. The opportunity cost of MTurk has been previously described [18]–[23]. Another design component was the HIT filter for the workers' approval rating. We restricted participation to workers with an approval rate of at least 90%, meaning that 90% or more of the participant's previous submissions were accepted by requesters. The worker's approval rating is a system of checks and balances within MTurks, where the quality of the worker's HITs either beneficially or adversely impacts their ability to complete future HITs [15]. This predetermined approval threshold, or inclusion criteria, promoted accuracy among completed questionnaires from the MTurk population without influencing selection bias. Last, to deter respondents from using search engines to assist their accuracy during the task and to encourage HIT completion in a single sitting, a time constraint was set on the HIT to a maximum of 10 minutes. We also included text within the questionnaire to clearly explicate that true answers were appreciated and that no participant would be penalized for incorrect responses, i.e. there would be no gain from looking for the correct answer.

After agreeing to participate in the HIT, workers began the task by reading through a brief introduction to the questionnaire, which included expectations of time to complete task and clear criteria for work acceptance or rejection. Exclusion criteria were determined as follows. Respondent surveys that were incomplete or offered multiple responses for the same question were rejected. To facilitate analysis, the MTurk website compiled participant responses and formatted them into a. CSV file which was easily downloaded into an Excel spreadsheet.

### Ethics Statement

The study involved the use of survey procedures obtained in such a manner that the human subjects could not be identified directly or through identifiers linked to the subjects and qualified as an exempt research activity under the Code of Federal Regulations [38 CFR 16.101(b) Section 3, Category 2].

## Analysis and Results

### Data Collection

Data collection was completed in 8 days (March 17th–25th, 2013), with 87% percent of subjects among the total sample pool meeting approval criteria for payment. Workers took 153.8 seconds on average to complete the survey. The working dataset is currently hosted on the website of the corresponding author, located at sr2c.case.edu/data.

### Participant Characteristics

**Figure 1:**
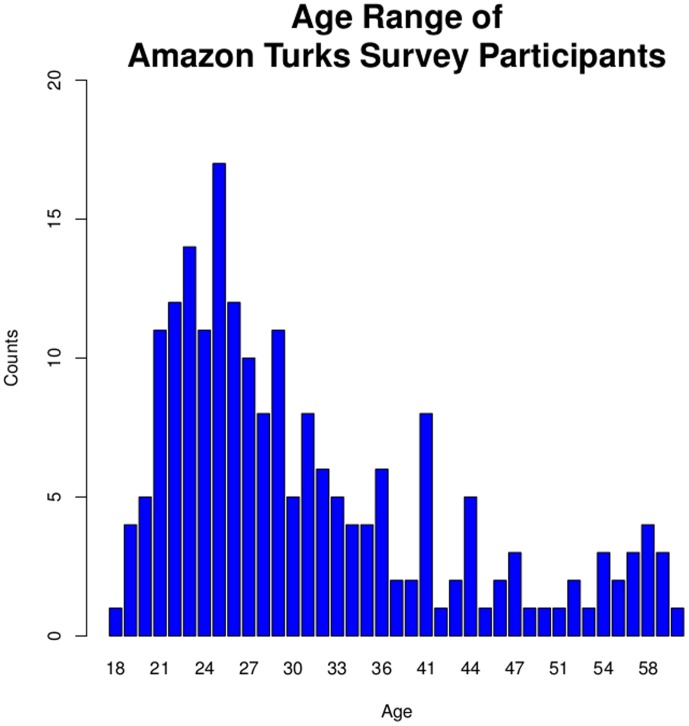
Age Distribution. The x-axis is age and y-axis is the frequency count. This representative population sample of 202 subjects was collected within 8 days. The median age was 28 years and the mean age was 32 years (SD = 10.79). 57% of respondents were male (N = 115), while 43% of respondents were female (N = 87).

232 eligible workers were initially enrolled in the study. 202 workers were in the final sample assessment. Twelve subject surveys were rejected for multiple response entries for the same question, and 10 subject surveys were rejected for missing a question entirely. In the final sample, ages ranged from 18 years to 61 years, with a median age of 28 and mean age of 32 years (SD = 10.79, Figure 1). 115 workers were male and 87 workers were female. The age distribution is slightly right skewed, similar to the current middle segment of the US population distribution [29], though shifting slightly toward a younger demographic. The lower truncation point was at 18 years due to minimal age requirement by MTurk, while the upper truncation point reflected the average senior citizens that were less technologically savvy [22].

### Analysis Strategy

In addition to age and gender, the data consisted of responses from a multiple choice survey questionnaire (Table 1). Therefore the EDA summary statistics are the counts and descriptive analyses of the categorical responses. Response counts were calculated based on cross-tabulation, ratio, and frequency table descriptions. Contingency table analyses were used to evaluate relationships between worker responses for ovarian cancer (the test element), and breast cancer (the control element). Multinomial logistic regression analyses were used to determine the outcome of knowledge background while controlling for age, gender, and cancer type. Correspondence analyses were used to examine the residual differences between the repeated measures element of the survey. Group ratios were compared using a Pearson's 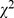 test and the exact multinomial test [30]. Trellis and correspondence analysis graphics were used for visualization of the results. Data were analyzed using R version 2.14.1 [31].

### Baseline Knowledge Analysis

**Figure 2:**
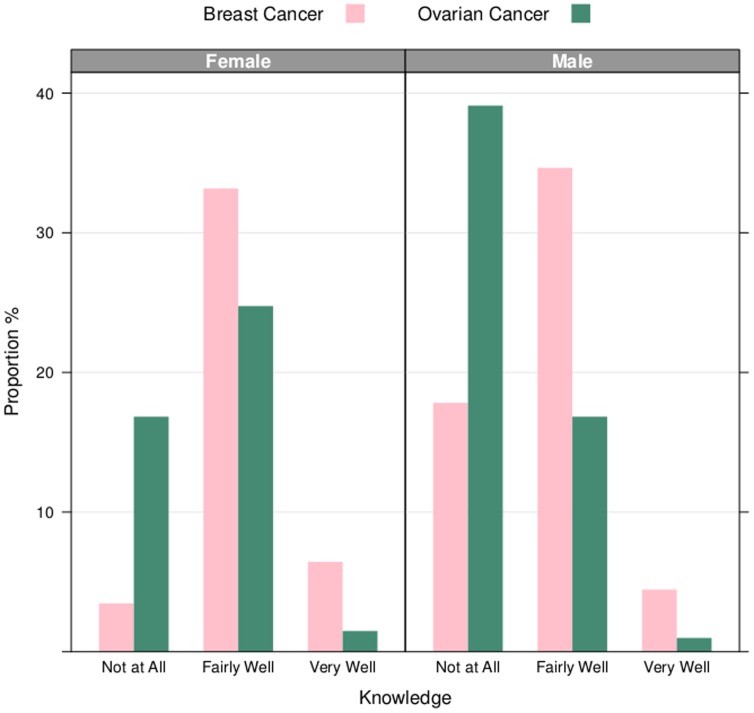
Gender Comparison of Diagnosis Risk Knowledge. The height of the bars are the proportion of men compared to women who knew of breast cancer or ovarian cancer “Not at All,” “Fairly Well,” or “Very Well,” respectively. Male participants presented a strong indication of breast cancer knowledge, as evidenced by the “Fairly Well” and “Very Well” categories. Conversely, male participants had virtually no knowledge of ovarian cancer. A majority of female participants also responded that they knew of ovarian cancer “Not at All,”however most of the female participants knew of ovarian and breast cancer “Fairly Well.” Both men and women did not know of breast cancer of ovarian cancer “Very Well.”

Table 2 shows a comparative projection of ovarian cancer knowledge versus breast cancer knowledge, based on cross tabulation of responses to Questions 3 and 4. Overall, the knowledge about ovarian and that of breast cancer were strongly and significantly different, with a p-value of 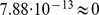, by the Pearson's Chi-squared test. Upon review of the specific differences, we found that 78.7% of all workers (N = (137+22)/202) reported they were “Fairly Well” or “Very Well” informed of breast cancer (Table 2). Conversely, over half, or 56% of workers (N = 113/202) reported *no knowledge* whatsoever of ovarian cancer. Individuals with prior knowledge of breast cancer tended to possess some knowledge of ovarian cancer: compare counts in Table 2 in the lower off-diagonal positions (70+18+1) vs those in the upper off-diagonal positions (1+2+0). Binomial comparisons of 3 individual categories between ovarian and breast cancer also showed significant differences, respectively, all with *p*<0.0014 using a 2-sample proportion test with continuity correction. The odds ratio was also strongly lopsided, with 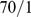 for the odds of “Not at All” to “Fairly Well” for ovarian cancer versus breast cancer; and 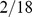 for the odds of “Very Well” to “Fairly Well” for ovarian cancer versus breast cancer (Figure 2).

**Table 2:** Contingency Table of Cancer Background Knowledge.

Variable	Ovarian Cancer			
Breast Cancer	Not at All	Fairly Well	Very Well	Total
Not at All	42	**1**	0	**43**
Fairly Well	**70**	65	**2**	137
Very Well	1	**18**	3	22
Total	**113**	84	5	202

The 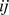-th entries are the number of participants who fall into the 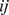-th categories. For example, 70 people responded that they knew of breast cancer “Fairly Well,” but knew nothing about ovarian cancer (“Not at All”). On the contrary, only one person claimed to know of ovarian cancer “Fairly Well,” but “Not at All” for breast cancer.

### Familiarity/Background Analysis

Based on responses to Questions 5 and 6, 12% of workers (N = 24/202) knew someone close to them who had been diagnosed with ovarian cancer. Knowledge of someone very close with a breast cancer diagnosis accounted for 46% (N = 92/202) of workers. The difference between the familiarity proportions of two cancers is obviously significant, at a p-value of 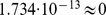, using 2-sample test for equality of proportions with continuity correction.

### Knowledge by Gender Analysis

We further explored knowledge of cancer on the condition of gender (Figure 2). Among the sample female population, 39% of women (N = 34/87) reported no knowledge of ovarian cancer; conversely 8% (N = 7/87) reported no knowledge of breast cancer. Almost 70% of men (N = 79/115) reported no knowledge of ovarian cancer; additionally 31% of men also reported no knowledge of breast cancer (N = 36/115). However, 92% of women reported fairly to very good knowledge of breast cancer (N = 80/87), while almost three-quarters of men reported fairly to very good knowledge of breast cancer (69%, N = 79/115). Proportions of knowledge by gender are visualized in Figure 2.

### Analysis of Multi-factor Impact on Knowledge

We extended these findings by determining which predictor variables, or covariates, contributed the most to the extent of cancer knowledge, and also examined the overall impact of multiple factors on the knowledge simultaneously. Therefore we conducted a multinomial logistic regression analysis. In this analysis, cancer knowledge is the polytomous response variable (call it 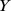), consisting of the categories: “Not at All,” “Fairly Well,” and “Very Well”, coded as 0, 1, 2. Age, Gender, and Cancer Type are the covariates denoted as 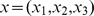. Here 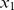 (*age*) is a continuous variable, 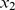 (*gender*) is a dichotomous variable coded as 1 and 0 for male and female, and 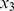 (cancer type) also coded as 1 and 0 for ovarian and breast cancer. Using the command *multinom* in the R package “nnet” [32], the resulting multinomial logistic regression relationship is
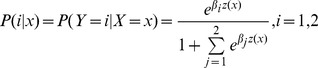
(1)for the “Fairly Well” and “Very Well” categories, and 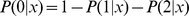 for the “Not at All” category. Here 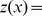 *(1, age, gender, cancer type, age⋅gender)'* was selected using the stepwise selection procedure based on AIC and represents an intercept term, linear effects in age, gender, and cancer type, as well as an interaction term between age and gender. The estimated coefficients and the associated one-sided p-values are given in Table 3. Thus, for the “Fairly Well” and “Very Well” categories, we have
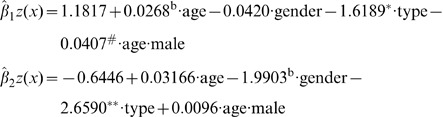
(2)where * indicates a statistically *extremely significant* coefficient with a p-value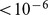, ^#^ indicates a *significant* coefficient with a p-value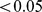, and ^b^ indicates a *slightly significant* coefficient with a p-value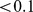 (Table 3). The equations (2) and p-values in Table 3 show clearly that the cancer type is the *most* significant factor in driving the difference in knowledge, with a severe drop by the negative coefficient for ovarian cancer (from breast cancer). The interaction of age and gender (male) acts as a somewhat secondary significant factor in driving the knowledge difference for the “Fairly Well” category, indicating that Knowledge about ovarian and breast cancers is less for older male, although older people (female) seemed to be more likely to know fairly well. For the “Very Well” category, again the cancer type is the most significant factor with a much smaller probability of people who'd know ovarian cancer very well than that for breast cancer. For the “Not At All” category, everything will be reversed: there is a sharp increase for being in the “Not At All” category about the ovarian cancer, as P(0) = 1-P(1)-P(2).

**Table 3:** Estimated Coefficients and Approximate 1-sided Significance.

		int	age	gender	typeov	age∶male
fairlywell	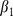	**1.1817**	**0.0268**	−0.0420	**−1.6189**	**−0.0407**
	p-value	0.0234	0.0558	0.4782	1.0997e-11	0.0421
verywell	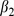	−0.6446	0.0317	**−1.9903**	**−2.6590**	0.0096
	p-value	0.2449	0.1084	0.0769	4.3918e-07	0.4028

Bolded coefficients are marginally to extremely significant from zero. Multiplying 1-sided p-values by 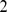 leads to 2-sided p-values. Results of this multinomial logistic regression determine that cancer type is the most significant factor related to cancer knowledge.

### Cancer Ribbon Color Awareness Analysis

We investigated public consciousness of the ovarian cancer awareness cause through identification of the national campaign's ribbon color. Table 1 summarizes the results from Questions 9 and 10, and shows that almost all responses correctly selected pink as the breast cancer awareness ribbon color (97.5%, N = 197/202). Furthermore by the secondary measure, i.e. responses to Question 9a, 94% of workers (N = 189/202) reported that their confidence in their responses were certain, or “100% sure” of their ribbon color choice for breast cancer. However, the responses for ovarian cancer ribbon color were more varied. Although over half of participants correctly selected the ovarian cancer ribbon color of teal (57%, N = 114/202), 19% of workers chose red (N = 39/202) as the correct ribbon color, 12% believed pink to also represent ovarian cancer (N = 25/202), while another 12% chose orange (N = 24/202) as a possibility. Also, by the secondary measure, the worker's confidence reveals that given just four colors, especially after a participant is sure about the color for breast cancer, only then can a significant portion of participants correctly guess the ovarian cancer ribbon color, with 93% of workers (N = 187/202) reported guessing, or being “50% sure” of their choice of ovarian cancer ribbon color.

### Cancer Risk Awareness Analysis

Based on Questions 7 and 8, the worker's best estimate of a woman's lifetime ovarian cancer risk is presented in Table 1. The response patterns indicated that 37% of workers (N = 75/202) correctly determined a woman's risk of ovarian cancer to be 1 in 70, and 38% of workers (N = 38/202) correctly determined a woman's risk of breast cancer to be 1 in 8. 27% of workers correctly identified both the lifetime risk of breast and ovarian cancer (N = 55/202). However, incorrect response pairs trended towards estimates of a woman's breast cancer risk to be 1 in 200 and ovarian cancer risk to be 1 in 1000 (20%, N = 41/202), or 1 in 70 for breast cancer and 1 in 200 for ovarian cancer (13%, N = 27/202). The worker's uncertainty revealed that 87% of responses (N = 175/202) were guesses for both ovarian and breast cancer risk.

### Lethality Knowledge Analysis

**Figure 3:**
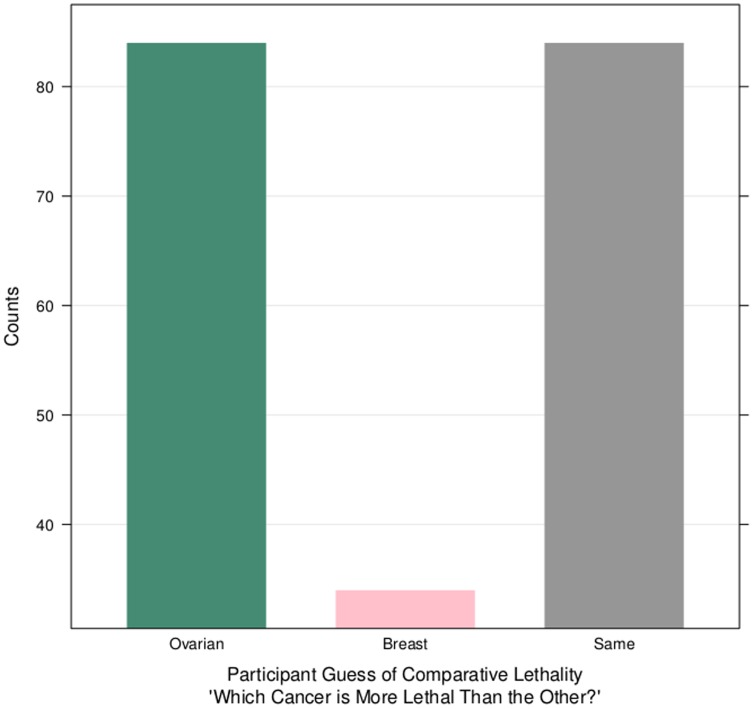
Knowledge of Lethality. The height of the bars are the frequency count of people who responded that ovarian cancer is more lethal, or breast cancer is more lethal, or they are of equal lethality. According to respondents, 16% believed breast cancer to be more lethal than ovarian cancer. 42% believed ovarian cancer to be more lethal than breast cancer, and 42% believed both cancers to be equally lethal. This indicates general lack of mortality knowledge, as the range of opinions varies widely.

Figure 3 and Table 1 articulate the workers' estimation of relative cancer death among ovarian and breast cancer, in their responses Question 11. Ovarian cancer is more lethal than breast cancer, yet 58% of respondents (N = 118/202) were incorrect in their assumptions, where *p* = 0.02. Specifically, responses revealed that 41.6% of workers (N = 84/202) correctly believed ovarian cancer to be more lethal than breast cancer and similarly, while 41.6% of workers (N = 84/202) falsely believed both cancers to be equally lethal to women, and the remaining 16.8% of workers (N = 34/202) also falsely believed breast cancer to be more lethal than ovarian cancer (Figure 3). Over three-quarters of workers reported guessing for their response to the cancer lethality survey question across all three answer choices (83%, N = 169/202).

### Knowledge Gap Analysis

**Figure 4:**
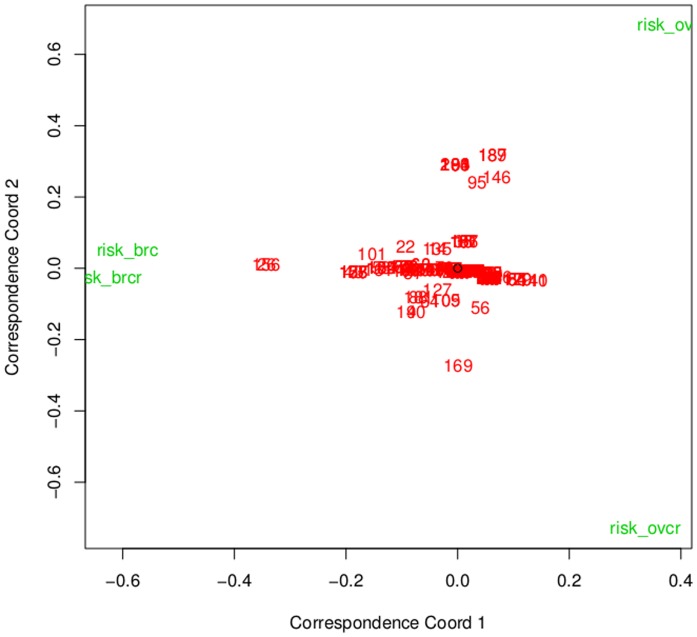
Correspondence Analysis of Risk. Correspondence is determined by distance away from horizontal and vertical axis. The cloud of raw data points in the middle of the graph have no relationship to one another and therefore are close to the horizontal and vertical midpoints of the graph. The points are further away from the vertical midpoint but close on the horizontal axis, which indicates a good relationship between the predictor variables. However, the points far away from the vertical midpoint and far away from the horizontal midpoint indicates a poor relationship. Therefore participants' selections for lifetime breast cancer risk were consistent in their initial and repeated survey responses. Responses for ovarian cancer lifetime risk varied widely, indicating that the participants were guessing their response selections.

Last, we examined the repeated measure residuals of the worker's responses for breast cancer and ovarian cancer risk (Figure 4). Correspondence analysis showed that workers were consistent in their responses concerning breast cancer risk, with little difference between the first time answering the question and the second time answering the question despite 88% (N = 178/202) admitting to guessing on that particular question. This was visualized by the leftmost points of Figure 4 which were very close. In contrast, the worker estimate of ovarian cancer risk varied considerably. in the first and 2nd attempts of their responses,as visualized in Figure 4 by the rightmost points in the graph. Like breast cancer, 97% (N = 195/202) of workers reported uncertainty in their estimate of ovarian cancer lifetime risk. This indicated the participant's incertitude and complete dearth of understanding of lifetime ovarian cancer risk (*p*<0.001).

## Discussion and Conclusions

Our study indicates that the US population consistently presents a lack of awareness of ovarian cancer impact or significance. However, individuals with prior knowledge of breast cancer tended to possess some knowledge of ovarian cancer. We unequivocally identified the knowledge gaps in a representative sample of the US population for both specific and generalized ovarian cancer information. Given that the distribution of ovarian cancer malignancies diagnosed at an early stage are fundamentally different than that of tumors present with advanced-stage disease [33], the findings underscore the importance and need for continuing efforts to improve awareness of ovarian cancer early diagnosis endeavors and promote its research.

This is the first study we know of among the US population on the awareness of ovarian cancer. A recent survey out of the University College of London, which surveyed exclusively United Kingdom women on their levels of symptom awareness, determined that women among their sample population were unable to recall any ovarian cancer symptoms [34], [35]. Their conclusions coincided with ours: that there is a severe knowledge gap among the general public about ovarian cancer and more research is needed for this devastating disease. Prior studies indicate that life experience, family history and stories about family history play a key role in constructed awareness of cancer risk among individuals from hereditary cancer families [33], [36]–[40]. The targeted population of our study, a reasonable proxy of the US population, does not necessarily possess this heuristic function of experiential ovarian cancer-related awareness to improve odds of early diagnosis. However, in future studies, it may be useful to quantify awareness of genetic risk and how it factors into public knowledge.

The current study design and implementation was performed to provide a unbiased framework with quality control and repeated measures elements. These strategies were constructed from a merger of clinical and theoretical perspectives. The cross-sectional, population-based design of the analyses were reliable, observed, and could be readily inferred.

Potential limitations in the design of the study include the MTurk worker pool. As of January 2013, MTurk no longer approves international accounts. The worker pool, once bolstered by global accounts of over 500,000 workers [41], is now restricted to individuals who must both reside in the United States and possess a valid social security number. It may be argued that MTurk workers are more technologically and Internet savvy, and have an age distribution that tends to be slightly younger than the general US population. This could unfairly bias results in general. However, given that technologically-knowledgeable internet users should be more knowledgeable, if they present a severe lack of knowledge of ovarian cancer then by extension the general US population should as well, thereby assisting our assertions [18]. Furthermore, American Amazon Turk workers have been evaluated thoroughly in the literature and are arguably closer to the US population as a whole than subjects recruited from traditional university subject pools. Last, conducting experimental research on MTurk offers benefits such as a low risk of dishonest responses, no risk of experimenter effects, and low susceptibility to coverage error in comparison to traditional studies [22].

Future studies employing our survey design will follow both specific and generalizable pathways. Greater specificity to ovarian cancer awareness will involve methodological extension as well as identification of distinct and specific early diagnostic symptom patterns. Broader application of this could involve extension of our survey design to other cancers with high mortality rates and vague symptoms, such as esophageal cancer and pancreatic cancer. Pancreatic cancer is the fourth most common cause of cancer-related death in the United States, and has an extremely poor prognosis of a 6% relative survival rate at Stage IV. Esophageal cancer, 3 to 4 times more common in men than women, also has a poor prognosis of a 3% relative survival rate at Stage IV. We advocate that the present study's survey methodology will be effective and efficient in its application beyond rare gynecological cancers, and will be particularly cost-effective for a nationwide assessment among patients diagnosed with low-prevalence diseases.
